# Network and functional analyses of differentially expressed genes in gastric cancer provide new biomarkers associated with disease pathogenesis

**DOI:** 10.1186/s43046-023-00164-5

**Published:** 2023-04-10

**Authors:** Mousa Fadaei, Maryam Kohansal, Omidreza Akbarpour, Mahsa Sami, Ali Ghanbariasad

**Affiliations:** 1 Noncommunicable Diseases Research Center, Fasa University of Medical Sciences, Fasa, Iran; 2 Department of Medical Biotechnology, Fasa University of Medical Sciences, Fasa, Iran; 3 Department of Biology, Payame Noor University, Tehran, Iran; 4 Genetic Laboratory, Fasa University of Medical Sciences, Fasa, Iran

**Keywords:** Gastric cancer, Gene expression, Signaling networks, Biomarkers

## Abstract

**Background:**

Gastric cancer is a dominant source of cancer-related death around the globe and a serious threat to human health. However, there are very few practical diagnostic approaches and biomarkers for the treatment of this complex disease.

**Methods:**

This study aimed to evaluate the association between differentially expressed genes (DEGs), which may function as potential biomarkers, and the diagnosis and treatment of gastric cancer (GC). We constructed a protein-protein interaction network from DEGs followed by network clustering. Members of the two most extensive modules went under the enrichment analysis. We introduced a number of hub genes and gene families playing essential roles in oncogenic pathways and the pathogenesis of gastric cancer. Enriched terms for Biological Process were obtained from the “GO” repository.

**Results:**

A total of 307 DEGs were identified between GC and their corresponding normal adjacent tissue samples in GSE63089 datasets, including 261 upregulated and 261 downregulated genes. The top five hub genes in the PPI network were CDK1, CCNB1, CCNA2, CDC20, and PBK. They are involved in focal adhesion formation, extracellular matrix remodeling, cell migration, survival signals, and cell proliferation. No significant survival result was found for these hub genes.

**Conclusions:**

Using comprehensive analysis and bioinformatics methods, important key pathways and pivotal genes related to GC progression were identified, potentially informing further studies and new therapeutic targets for GC treatment.

**Supplementary Information:**

The online version contains supplementary material available at 10.1186/s43046-023-00164-5.

## Background

Gastric cancer is a common form of cancer with the second-highest cancer-related mortality rate [1]. More than 90% of gastric tumors are adenocarcinoma, and there is a poor prognosis for that. Early stages of the disease are often silent, so late diagnosis results in a low survival rate [2]. In spite of the improvement in the diagnosis of gastric tumors and the development of new molecular targeted drugs, there is still a lack of diagnostic biomarkers and effective treatments [3, 4].

In recent years, the development of bioinformatics methods and tools has made significant progress at determining the molecular pathogenesis of many carcinomas and adenocarcinomas such as breast and gastric cancer [5, 6]. One way to identify biomarkers in a biological context is by analyzing gene expression transcriptomic data [7]. Expression of many genes is deregulated once cells start to transform toward a cancerous phenotype, and this is different from cell to cell and tissue to tissue [8, 9]. As a result, analyzing the differentially expressed genes (DEGs) in a specific biological context like gastric cancer enables us to find potential diagnostic biomarkers and therapeutic genetic targets. For instance, CXCL1, SPARC, SPP1, and SULF1 are the genes overexpressed together in gastric cancer [10]. In another study, they proposed seventeen genes differentially expressed between gastric cancer samples and paired normal samples responsible for tumorigenesis [11]. Upregulation of CCNE1 and downregulation of NR3C1 is an indicator of primary GC tumor, while downregulation of NR4A2 and upregulation of HSP90AA1 are promising markers of liver metastasis [12].

In the present study, a gene expression microarray dataset with GSE63089 accession ID was downloaded from Gene Expression Omnibus (GEO) database. There were two groups of normal and gastric cancer samples in the dataset statistically compared to identify genes different in expression between the two groups. The goal was to recognize new biomarkers among a great extent of DEGs with desired thresholds by the tools of network analysis and gene set enrichment analysis. Several hub genes and gene families engaged in biological processes and signaling pathways related to cancer progression were introduced, and their molecular mechanism leading to GC progression was explained.

## Results

### Data preprocessing

**Fig. 1:**
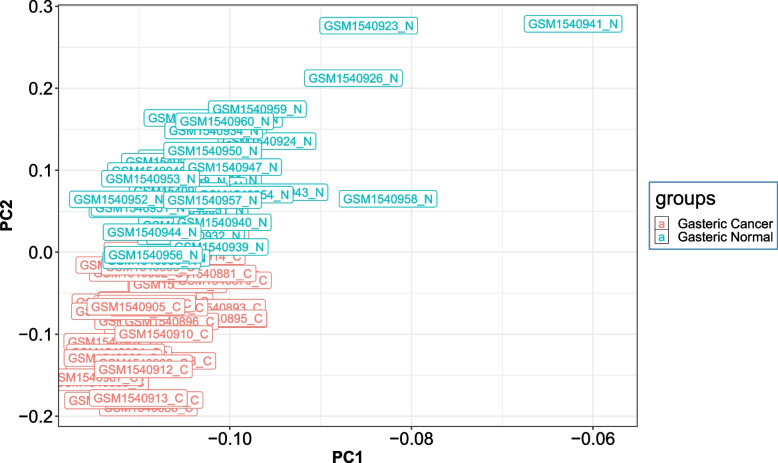
Illustration of outlier samples in the PCA plot. PC1 is the eigenvector one, and PC2 is the eigenvector two. Normal samples are blue with the suffix N, while tumor samples are red with the suffix C. one of the apparent outlier samples is GSM1540941_N in the top right corner

**Fig. 2:**
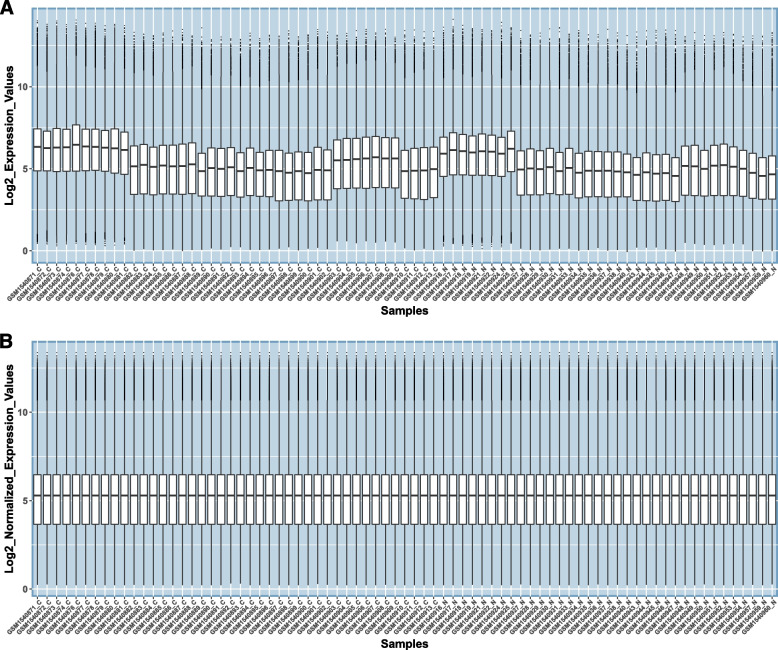
Boxplots before (**A**) and after normalization (**B**). Sample names with the suffix N are normal, and the ones with the suffix C are cancerous. Values were log2 transformed. Quantile normalization equalized the percentiles of all samples

The gastric cancer gene expression dataset was imported into R using “getGEO” function in “GEOquery” R package. Data were visualized using PCA and boxplots to recognize biased samples. Figure 1 illustrates the PCA plot for all samples before outlier sample removal. Two clusters appeared in the PCA plane segregated well based on the group definition. A number of samples were located at a distance from their cluster set regarded as the outlier or biased samples. Figure 2A depicts the sample boxplots before outlier removal. Samples with extreme IQRs demonstrated the presence of batch effects in the dataset, and they were removed as well. As a result, outlier samples could no longer impact the downstream processing steps. Thirteen outliers were detected, presented in Supplementary file 1. Next, data were normalized using “normalize.quantiles” function in “preprocessCore” R package. Figure 2B depicts sample boxplots after data normalization.

### Identifying differentially expressed genes

The cancer group was compared to the normal group in the dataset. There were 42 samples in the cancer group and 35 samples in the normal group. Genes with absolute log fold change (LogFC) larger than one and adjusted *p*-value less than 0.05 were regarded as DEGs. 261 upregulated genes and 216 downregulated genes were recognized. The mentioned metrics for all DEGs are presented in Supplementary file 2. SFRP4, CDH17, FAP, CLDN1, and OLFM4 were of the highest LogFC values among the upregulated genes, while GIF, PGA4, GKN1, ATP4B, and CPA2 had the lowest LogFC values among the downregulated genes.

### Undirected protein-protein interaction network

**Fig. 3:**
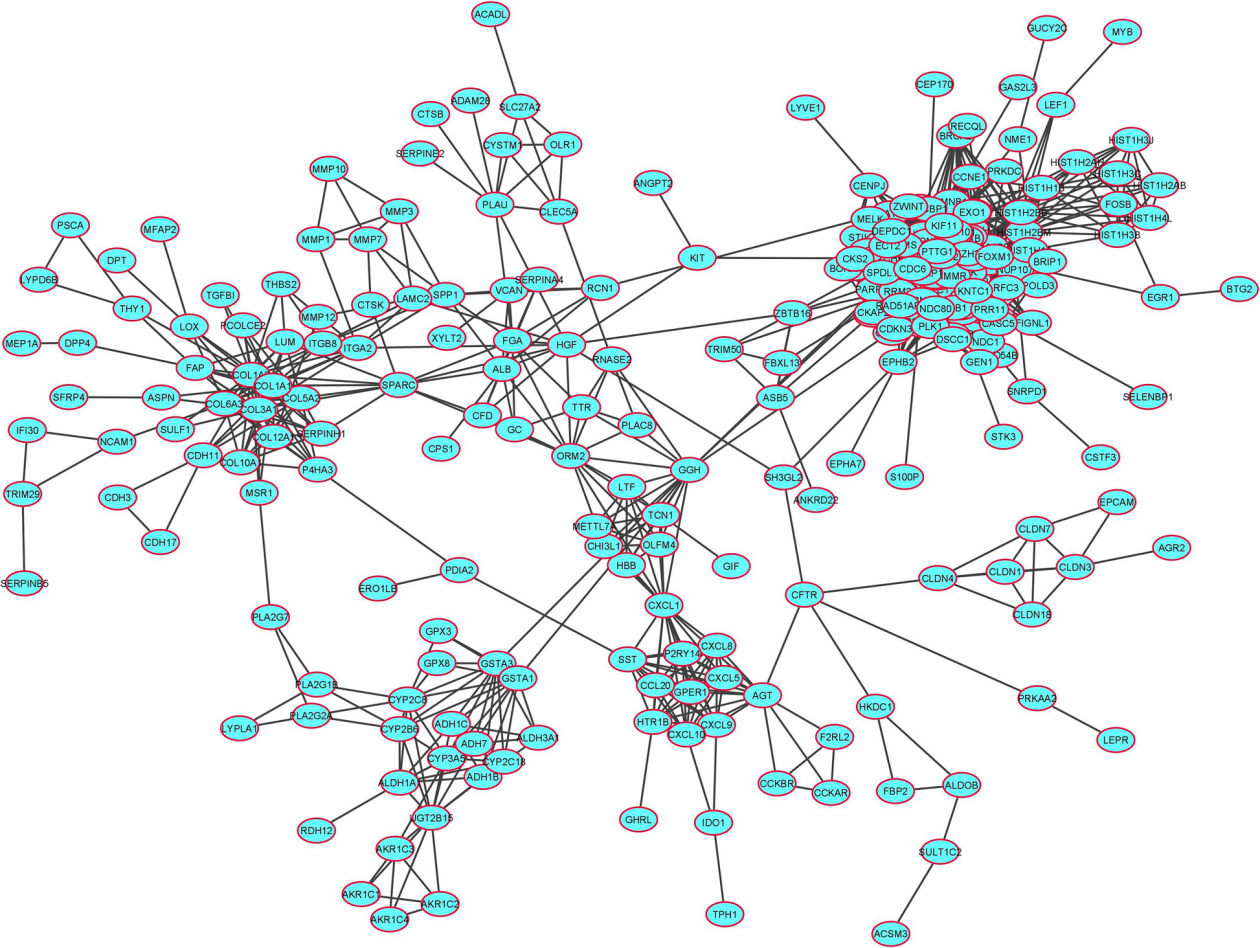
The whole network giant component. Labels are protein/gene symbols. This is a scale-free network [13] that follows a power-law distribution (most network nodes have a low degree while there are few nodes with a high degree)

All DEGs were given to the STRING database to construct the protein-protein interaction (PPI) network. Three sources of evidence were used to predict more valid interactions between genes, namely Experiment, Database, and Co-expression. 307 DEGs were identified to have interaction with at least one gene (protein) that participated in the network configuration. The giant component of this network with 268 nodes and 3582 edges is illustrated in Fig. 3. At one glance, there is a cluster of genes on the right-hand side of the network that may be responsible for one or some specific biological functions. Therefore, we decided to apply cluster analysis on the network and separately carry out the enrichment analysis on each cluster.

### Network descriptive and hub genes

The network diameter was Eleven containing AKR1C1, AKR1C3, CYP3A5, GSTA, GGH, CXCL,1AGT, CFTR, HKDC1, ALDOB, SULT1C2, and ACSM3 genes. Transitivity was around 80%, edge density was about 10% and the mean distance was 4.2. Two important centralities, degree and betweenness, and regulation status for some genes with high centrality are presented in Table 1. CDK1 had the highest value for both degree and betweenness centralities upregulated in our analysis. HGF had a high betweenness but a low degree. Contrary, CCNB1 and CCNA2 had a high degree but low betweenness. Other descriptives such as edge-betweenness for edges and closeness and average distances for each node are provided in Supplementary file 3.

**Table 1:** The network hub genes. DEGs were sorted based on the highest degree. Status column exhibits whether a gene is upregulated or downregulated in the analysis

De genes	Degree	Betweenness	Status
cdk1	102	6366.23	Up
CCNB1	94	324.32	Up
CCNA2	93	476.87	Up
CDC20	89	1735.14	Up
PBK	87	2489.09	Up
CHEK1	80	1230.53	Up
ZWINT	79	2548.27	Up
CDKN3	72	1791.96	Up
HIST1H2BB	25	747.8	Up
HIST1H2BM	25	747.8	Up
COL1A2	21	2108.26	Up
COL1A1	19	1422	Up
COL3A1	18	1345.35	Up
CXCL1	18	4174.93	Up
ORM2	15	3172.16	Down
EPHB2	14	2414.36	Up
AGT	14	1555.01	Up
FGA	12	1101.23	Down
SPARC	12	4279.68	Up
GSTA1	12	2290.52	Up
GSTA3	12	2290.52	Down
ITGA2	11	2797.82	Up
HGF	11	6349.3	Down
ZBTB16	7	3430.08	Down
RCN1	5	2436.24	Up
CFTR	5	3687.92	Up

### Network clustering and enrichment analysis

**Fig. 4:**
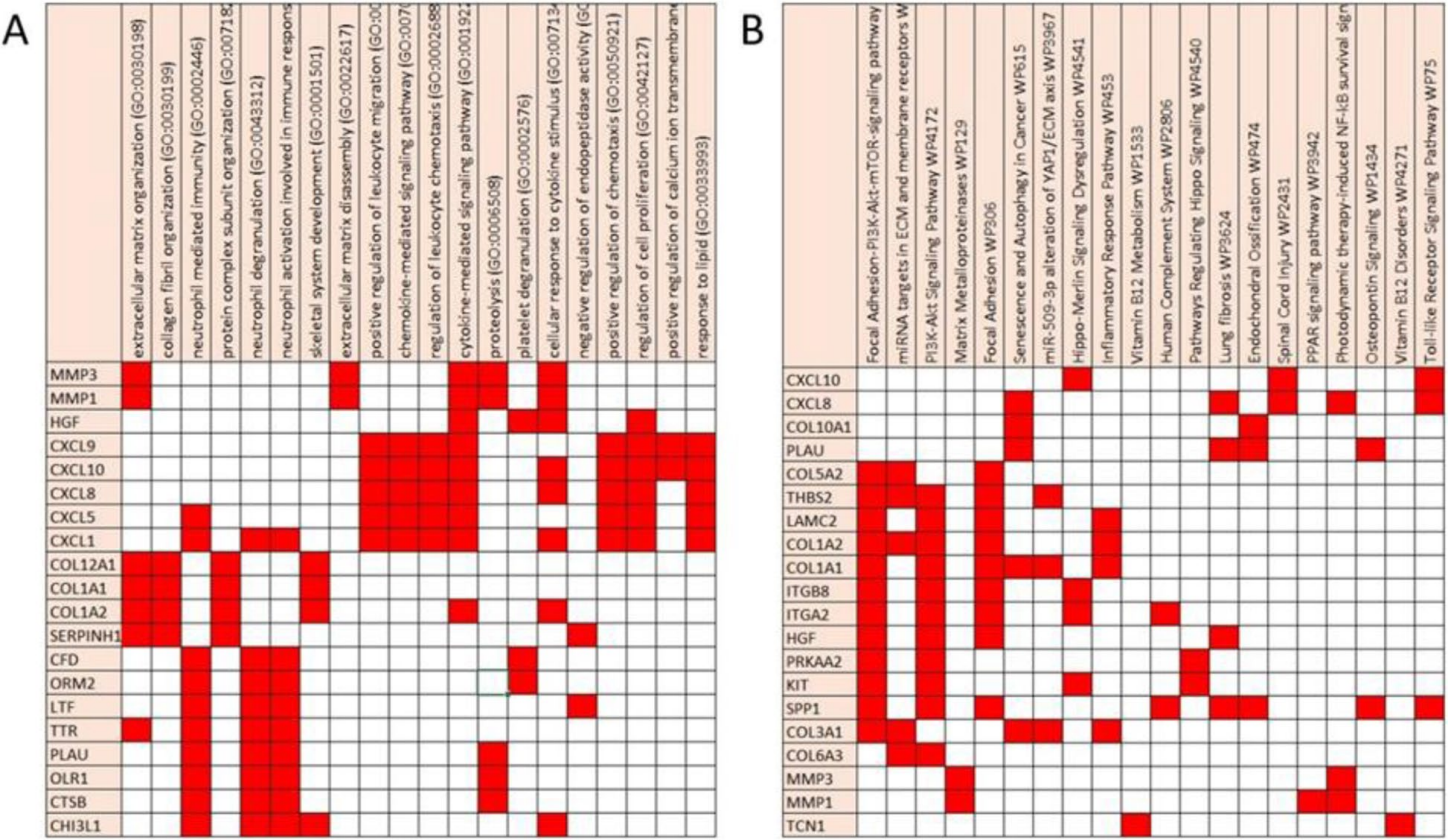
Gene Set Enrichment Analysis for cluster 1 of the PPI network. Part A illustrates the enriched terms for biological process from GO repository. Part B shows the enriched pathways from WikiPathways signaling datasets. Red cells reveal the involvement of the genes in the enriched elements. Enriched terms were sorted based on the highest *p*-value

Network giant component was clustered using the “Fast Greedy” algorithm in “igraph” R package. Six clusters emerged and Gene Set Enrichment Analysis (GSEA) was performed on the two largest groups. Gene sets were given to the “Enrichr” online tool. Figure 4A and B depict the enrichment results for the first module. Enrichment results for cluster 1 are presented in Fig. 4 while results for cluster 2 are presented in Supplementary file 4. In Fig. 4A, the first two genes, MMP1 and MMP3, were related to the important terms for extracellular matrix (ECM) organization and degradation. Moreover, the CXCL gene family owned a number of terms associated with cytokine- and chemokine-mediated leukocyte migration. Collagen gene family was associated with ECM collagen fibers organization, but MMP1 and MMP3 genes were enriched for ECM remodeling proposing that extracellular matrix in gastric tumor samples has been altered probably in favor of the tumor. The last genes were bolding some Biological processes related to neutrophils activation and immune system. In cluster 2, “tubulin-binding” had the highest *p*-value among the biological processes. In addition, Kinesins (KIF) gene family was enriched for several terms associated with microtubules activity and organization as well as Kinase and ATPase activities. Furthermore, “DNA-dependent ATPase activity” and “DNA binding” terms were related to the same genes.

In B, some crucial terms for focal adhesion arrangement and PI3K-AKT signaling pathways were associated with many genes such as collagens, integrins, HGF, and so on. Moreover, matrix metalloproteinase-linked enriched pathways were associated with MMP1 and MMP3, similar to GO biological process terms. As a result, the role of obtained terms in gastric cancer should be interrogated. Some genes were involved in retinoblastoma protein signaling in cluster 2. A number of enriched genes encompassed terms related to DNA damage and repair, proposing that DNA damage might have occurred in tumor cells. RFC3, TOP2A, and FANCI were the genes linked to the signaling pathways engaged in Gastric Cancer Network 1 and 2.

### Gene expression validation

**Fig. 5:**
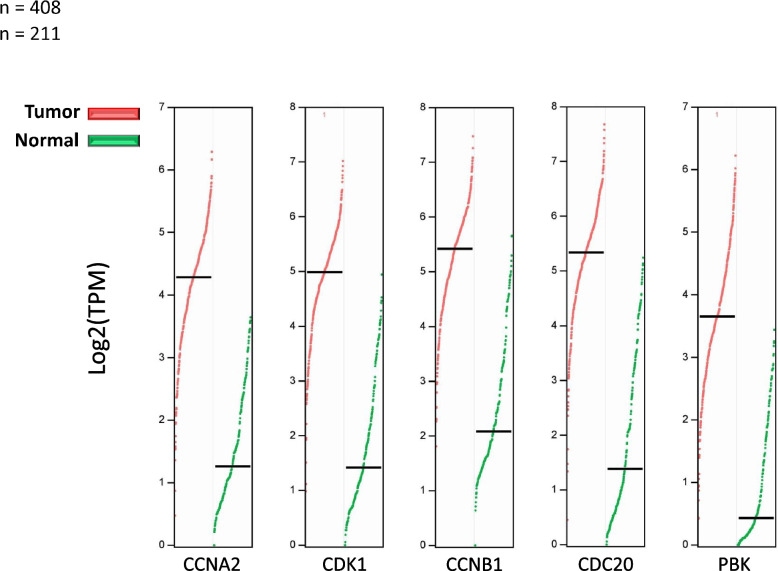
Expression profiling of the hub genes. Data were firstly TPM normalized and then log2 transformed. The median for all the hub genes in gastric tumor samples was larger than in normal samples

Survival analysis was performed on the enriched genes in Fig. 5. To verify the expression and impact of the genes on survival rate. Collagen gene families were the dominant protein family in the enrichment analysis, some of which had a significant *p*-value, < 0.05. Patients with higher expression levels of collagens in Fig. 4, had lower daily survival rates presented in Supplementary file 5. However, the results for the hub genes were not significant therefore, we only validated them using expression profiles in TCGA and GTEx datasets. Hence, upregulation of all the top five hub genes in Table 1 was verified in Fig. 5.

## Discussion

Gastric cancer is still the fourth most common cancer globally and ranks as the second leading cause of cancer death alongside lung cancer [14]. GC is a complex disease affected by many environmental and genetic factors. Despite the increase in knowledge and advances in drug development, treatment still performs poorly due to late diagnosis and extremely high heterogeneity within the tumor and among patients. Therefore, there is an urgent need to identify more specific and sensitive biomarkers to clarify this complex disease’s pathogenesis. These include the telomerase reverse transcriptase promoter region (TP53, BRAF, and RAS), DNA hypermethylation, and other gene mutations that can be used to explore the pathogenesis of gastric cancer.

In our study, several hub genes as well as outcomes for functional analysis went under investigation. As a result of tumor heterogeneity, gene expression profiles (GSE) emerged from multiple modules from protein-protein interaction information. The largest (first) module enriched mostly for the immune system, tumor occurrence, and progression. Components of this cluster are engaged in focal adhesion formation, ECM remodeling, and cell migration. Some common cancer signaling pathways such as PI3K-Akt, chemokine-mediated signaling pathway, ECM-receptor interaction, and so on emerged [15–17]. The ECM-receptor interaction pathway also plays a vital role in the proliferation, differentiation, and metastasis of cancer cells [18]. ECM can promote cancer metastasis by inducing epithelial-mesenchymal transition (EMT) of tumor cells [19]. integrins, collagens, and matrix metalloproteinases were among the important DEGs responsible for de novo remodeling of ECM and the stiffness pertinent for EMT mechanism and cell migration [20–22]. Integrin signals bridge between cell ECM and cell differentiation [23]. Survival analysis shows that all collagens enriched in our study play a pivotal role in integrin signaling since they are against the survival rate of gastric cancer patients. The majority of them even had a significant *p*-value demonstrating the significance of collagens upregulation in GC progression. These collagens were enriched in the PI3K-Akt signaling pathway engaged in focal adhesion formation. Furthermore, The PI3K/Akt survival signals regulate gene expression and cell metabolism, and the reaction products catalyzed by PI3K essential for adhesion, survival, cytoskeleton rearrangement, and vesicle transport [24]. The control of cell growth by the PI3K/Akt pathway via regulating cell proliferation, cell cycle progression, and apoptosis implicates a crucial role of this pathway in carcinogenesis and cancer development [24, 25]. Therefore, regulating ECM and PI3K1/AKT signaling pathways appear to be promising treatment strategies [24]. There were some CXC chemokine ligands (CXCL) in cluster 1 as well. They have important roles in lymphocyte trafficking, particularly the attraction of leukocytes to tumor sites, induction of apoptosis, regulation of cell growth, and mediation of angiogenesis [26, 27]. All the CXCL genes were upregulated in our analysis increasing ligands for chemokine receptors that triggers cell migration of tumor cells [27]. CXCL1 was among the hub genes and possessed a relatively significant *p*-value in survival analysis. The higher this gene is expressed, the survival rates is reduced in GC patients.

The top five hub genes in the PPI network CDK1, CCNB1, CCNA2, CDC20, and PBK, exhibited unique expression Patterns. These DEGs and related functions would be related to the progression of gastric cancer. However, no significant survival result was found for these hub genes. We validated them by expression profiling of these genes in TCGA and GTEx genomic repositories rather than the former analysis.

Cdk1 has a catalytic subunit that promotes the M-phase process essential for the G1/S and G2/M phase transitions during cell proliferation [28, 29]. Besides, cyclin B-Cdk1 is involved in cell survival at mitotic checkpoints called spindle checkpoints [30, 31]. In the past ten years, a large number of studies have shown that the disorder of CDK1 not only leads to the rapid growth of tumors but also leads to the spontaneous proliferation of cancer cells [32, 33]. Prior studies have indicated that aberrant activation of CDKs and their modulators exist in many tumors [34]. Neganova et al. reported that CDK1 is a critical element of cell cycle regulation, especially mitosis, and plays a vital role in maintaining the pluripotency and genome stability of human pluripotent stem cells [35]. In addition, CDK1 can activate the JAK/STAT3 signaling pathway through the phosphorylation of JAK1, thereby promoting the progression of colorectal cancer (CRC) [36].

Cancer is characterized by cell cycle dysregulation. CCNB1, Cycline B1, is one of the central genes in the first module of the PPI network whose product promotes the transition of cells from the G2 to M phase [37]. CCNB1 depletion or stable gene silencing of CCNB1 can inhibit human tumor cell proliferation and induce apoptosis [38]. Related studies have shown that the overexpression of CCNB1 is connected to the clinical stage, lymph node metastasis, and low survival rate of GC patients [39]. Furthermore, CDK1 and CCNB1 are highly expressed in neuroblastoma (NB) cell lines enhancing their proliferation [40]. The important role of the CDK1/CCNB1 complex in tumor cell survival was confirmed [34]. Finally, A recent study reported that cyclin B1 and cyclin B2 are the most important candidate biomarkers in GC [41].

Like CCNB1, Cyclin A2 (CCNA2) is another hub gene a member of the cyclin family that functions as a regulator of cyclin-dependent kinases (CDKs) affected by KRAS gene mutations. It is also a predictive biomarker of gastric cancer associated with Polo-like kinase 1 (PLK1), a mitotic serine/threonine-protein kinase [42]. Cyclin B1 and Cyclin A2 form a complex with CDK1 to trigger G2/M transition [43]. Upregulation of CCNA2 is found in numerous types of cancer, including pancreatic ductal adenocarcinoma and colorectal cancers [44, 45]. CCNA2 has the potential to be a new diagnostic biomarker and cancer therapy target that aims to monitor the efficacy of breast cancer chemotherapy [46]. Furthermore, these two genes were present in cluster 2 of the PPI network enriched alongside Kinesin gene family (KIF). KIF proteins are the hub proteins in the intracellular transport system by transport of cellular cargo. Mitotic spindle kinesins are essential for cell division. Therefore, genes in cluster 2 were engaged mostly in cellular proliferation, such as TOP2A and RFC3. Furthermore, many non-mitotic kinesins are associated with tumorigenesis and anticancer drug resistance [47].

Cell division cycle 20 homolog (CDC20) is an oncogenic gene that has long been recognized as one of the significant regulatory components of the cell cycle [48, 49]. Its product forms a complex with Anaphase Promoting Complex (APC) necessary for spindle assembly and chromosome segregation [50]. Overexpression of CDC20 has been reported in various malignant tumors [51]. It has been proposed to be a promising therapeutic target for cancer treatment as well [50]. The high expression of CDC20 is associated with increased tumor grade and stage in the majority of common carcinoma and adenocarcinoma [52]. P53 negatively regulates CDC20 expression, and silencing of CDC20 significantly inhibits cell growth in vitro [53]. Inactivation of p53 has been observed in various cancer tissues, including acute myeloid leukemia and lung cancer [54–56]. This effect might be attributed to CDC20 upregulation in gastric cancer. APC/CDC20 complex can suppress apoptosis by targeting the apoptotic protein BIM for destruction and ubiquitination [57, 58].

PBK is a serine/threonine-protein kinase related to the mitogen-activated protein kinase kinase (MAPKK) family. Overexpression of this gene has been implicated in tumorigenesis [59, 60]. PBK gene is upregulated in various types of cancers and tumors such as bladder cancer, brain tumor, breast cancer, hepatocarcinoma, lung cancer, and gastric cancer [61–65]. PBK is also located in the nucleus and is involved in the phosphorylation of histone H3 and the inhibition of p53 in colorectal and breast cancer cells in different cancers [66]. PBK is connected to geranylgeranylation signaling, most likely in advanced-stage cancers [67], which is essential for cancer cell proliferation, confirming that PBK is an important molecular target for cancer therapy.

## Conclusion

In conclusion, chemokine ligands, integrins, collagens, and matrix metalloproteinases, and hub genes including CDK1, CCNB1, CCNA2, CDC20, and PBK have been identified to be associated with GC progression. Through GO and pathway enrichment of the two most extensive modules, we identified the functions and pathways of the hub genes as well as genes responsible for cell differentiation and migration in cluster 1 and the ones responsible for cell cycle progression in cluster 2. In vitro studies are further required to test the functional results that pave a prospective way towards gastric cancer treatment.

## Methods

### Database searching to find a suitable experiment

Gene Expression Omnibus (http://www.ncbi.nlm.nih.gov/geo/) database was searched to detect an experiment containing high-quality transcriptomic samples in concordance to the study design. Searches were filtered for Homo sapiens, while gastric cancer and metastasis were the search keywords. Microarray raw data with accession numbers GSE63089 was selected and the gene expression matrix was downloaded from GEO using “GEOquery” R package version 2.5 [68]. The dataset contained 45 normal samples and 45 samples from gastric cancer patients. All the sample IDs are listed in Supplementary file 1.

### Identifying differentially expressed genes

Outlier samples were identified and removed using the PCA method. Next, data were normalized using the quantile normalization method [69]. GPL5175 annotation dataset was downloaded from GEO, and annotation was carried out by mapping probesets to the gene symbols. “Limma” R package, which applies linear models on the expression matrix, was utilized to discover DEGs between three groups of samples [70]. Genes with absolute log fold change larger than 1, and Benjamini Hochberg adjusted *p*-value [71] less than 0.05 were selected as the DEGs.

### Network construction

STRING database was used to generate the Interactions between all DEGs according to five sources of evidence, namely Experiments, Databases, Co-expression, Gene fusion, and Co-occurrence. Using “igraph” package version 1.2.4 in R software [72], the giant component of the network was extracted from the whole network. Next, different network descriptive and centralities were computed employing the same package.

### Enrichment analysis

Enrichment analysis was performed using the “Enrichr” online tool [73]. Enriched terms for Biological Process were obtained from the “GO” repository. For pathway enrichment analysis, the “wikiPathways” signaling repository version 2019 for humans was used. Enriched terms with the top score and a *p*-value less than 0.05 were selected.

### Survival analysis and expression profiling

Genes were given to the “GEPIA2” web server, and TCGA and GTEx gene expression data from stomach adenocarcinoma were employed for survival analysis and expression profiling [74, 75]. Kaplan-Meier estimate was used to perform survival analysis [76]. Overall survival was measured based on days with a 95% confidence interval, and the median was used as the cutoff for grouping. For expression profiling, the LogFC cut-off was set on one and *q-*value on 0.01. Data were Log2 transformed, and the “LIMMA” method was used for the statistical inference.

## Supplementary Information

**Additional file 1.**

## Data Availability

Anybody can access the GEO database that uses in this study with the below link: https://www.ncbi.nlm.nih.gov/geo/query/acc.cgi?acc=GSE63089.

## Abbreviations

DEGs: Differentially expressed genes
GC: Gastric cancer
PPI: Protein-protein interaction
GSEA: Gene Set Enrichment Analysis
ECM: Extracellular matrix
GSE: Gene expression profiles
EMT: Epithelial-mesenchymal transition
CXCL: CXC chemokine ligands
CCNA2: Cyclin A2
PLK1: Polo-like kinase 1
KIF: Kinesin gene family
CDC20: Cell division cycle 20 homolog
MAPKK: Mitogen-activated protein kinase kinase
